# Breath-print analysis by e-nose for classifying and monitoring chronic liver disease: a proof-of-concept study

**DOI:** 10.1038/srep25337

**Published:** 2016-05-05

**Authors:** Antonio De Vincentis, Giorgio Pennazza, Marco Santonico, Umberto Vespasiani-Gentilucci, Giovanni Galati, Paolo Gallo, Chiara Vernile, Claudio Pedone, Raffaele Antonelli Incalzi, Antonio Picardi

**Affiliations:** 1 Clinical Medicine and Hepatology Department, Campus Bio-Medico University, via Alvaro del Portillo 200, 00128 Rome, Italy; 2 Center for Integrated Research - CIR, Unit of Electronics for Sensor Systems, Campus Bio-Medico University, via Alvaro del Portillo 200, 00128 Rome, Italy; 3 Chair of Geriatrics, Unit of Respiratory Pathophysiology, Campus Bio-Medico University, via Alvaro del Portillo 200, 00128 Rome, Italy; 4 San Raffaele- Cittadella della Carità Foundation, Taranto, Italy

## Abstract

Since the liver plays a key metabolic role, volatile organic compounds in the exhaled breath might change with type and severity of chronic liver disease (CLD). In this study we analysed breath-prints (BPs) of 65 patients with liver cirrhosis (LC), 39 with non-cirrhotic CLD (NC-CLD) and 56 healthy controls by the e-nose. Distinctive BPs characterized LC, NC-CLD and healthy controls, and, among LC patients, the different Child-Pugh classes (sensitivity 86.2% and specificity 98.2% for CLD *vs* healthy controls, and 87.5% and 69.2% for LC *vs* NC-CLD). Moreover, the area under the BP profile, derived from radar-plot representation of BPs, showed an area under the ROC curve of 0.84 (95% CI 0.76–0.91) for CLD, of 0.76 (95% CI 0.66–0.85) for LC, and of 0.70 (95% CI 0.55–0.81) for decompensated LC. By applying the cut-off values of 862 and 812, LC and decompensated LC could be predicted with high accuracy (PPV 96.6% and 88.5%, respectively). These results are proof-of-concept that the e-nose could be a valid non-invasive instrument for characterizing CLD and monitoring hepatic function over time. The observed classificatory properties might be further improved by refining stage-specific breath-prints and considering the impact of comorbidities in a larger series of patients.

The characteristic sweet, musty and slightly faecal aroma of the exhaled breath, termed *fetor hepaticus,* has always been considered a hallmark of the patient with liver insufficiency. Liver plays a key role in metabolism and even early stages of hepatocellular failure, or the formation of porto-systemic veno-venous shunts secondary to portal hypertension, can alter the blood concentration of various endogenous intermediates, which, if volatile, can be analysed in the exhaled breath.

In the last decades, thanks to the development of sensitive analytical techniques, such as gas-chromatography/mass-spectrometry (GC/MS) or ion mobility spectrometry/multicapillary column (IMS/MCC), the clinical utility of breath analysis was evaluated for different diseases as, for instance, screening for lung and colorectal cancer and for chronic obstructive pulmonary disease (COPD)^1^,^2^,^3^,^4^,^5^,^6^. Accordingly, few information were also collected on the spectrum of volatile organic compounds (VOCs) in liver disease^7^,^8^,^9^,^10^,^11^,^12^,^13^. For instance, Van der Velde *et al.* found 4 VOCs (three ketones and one sulphur compound) able to significantly distinguish cirrhotic patients from healthy controls^7^ and, later, Morisco *et al.*, using a more sensitive technique (Proton Transfer Reaction Time-of-Flight Mass Spectrometry–PTR-MS), managed to find 12 VOCs (four ketones, two terpenes, four S and N containing compounds and 2 alcohols) significantly associated with LC and also with different Child-Pugh classes (CPC)^8^. To note, all these works aimed at identifying specific compounds associated with liver disease using an analytical approach, which is able to recognize the chemical nature of single VOCs, but lacks of synthetic properties. Hence, it cannot represent the global complexity of all VOCs present in the exhaled breath and, furthermore, it is expensive and time consuming.

The electronic nose (e-nose) technology is a novel technique, that consist of a gas sensor array and provides a sort of fingerprint of exhaled breath (breath-print, BP) by detecting VOCs through multiple sensors. In this case individual VOCs of exhaled breath profiles remain unidentified, but the e-nose is able to give a comprehensive VOCs profile, that has been shown to distinguish cancer from non cancer respiratory patients as if lung cancer were associated with the release of distinctive VOCs^14^,^15^. The e-nose has also been able to separate asthmatics from healthy controls^16^ and from COPD patients, based on well distinguished exhaled breath patterns, likely reflecting the well-known differences in pathogenic mechanisms of asthma and COPD^17^. All together, these findings suggest that exhaled breath qualifies as a sort of BP of selected diseases, and, thus, might be useful for diagnostic purposes as well as to monitor the response to therapy.

Unfortunately, so far, there are no studies on patients with chronic liver disease (CLD) with e-nose.

In patients with CLD, further than the staging of fibrosis in the pre-cirrhotic phase, there are two clinically relevant end points: the detection of LC (screening for esophageal varices and for hepatocellular carcinoma), which is currently performed by liver biopsy or on a clinical base; the evaluation of liver function in patients with LC (prognosis, referral for liver transplantation), which is assessed by clinical scores (Child-Pugh and MELD scores). Actually, liver biopsy is an invasive procedure and can be affected by sampling bias, while clinical and biochemical assessment and monitoring of LC lack the desirable diagnostic accuracy and suffer the fluctuations typical of CLD. Therefore, alternative diagnostic methods able to achieve these goals are eagerly awaited.

The aim of this proof of concept study was to assess discriminative and classificatory properties of the e-nose in CLD by comparing the BPs of patients with and without LC and hepatocellular failure. Verifying whether VOCs pattern changes depending upon the infective or non-infective origin of the liver disease represents a secondary outcome of the study.

## Methods

### Study participants

In order to perform an exploratory study, 104 consecutive patients with CLD and 56 healthy subjects were enrolled in the study. Patients with CLD were further classified into two groups according to the following criteria: 1) the group of patients with non-cirrhotic chronic liver disease (NC-CLD), including patients with a) ultrasound-documented hepatic steatosis with/without alcohol abuse; b) chronic viral hepatitis B or C; c) chronic autoimmune hepatitis, without clinical, biochemical or ultrasonographic signs compatible with the diagnosis of LC; 2) the group of patients with LC, including patients with CLD from different aetiologies and with the histological or clinical diagnosis of LC.

All patients and healthy subjects received further diagnostic evaluation by blood testing, including liver and kidney function tests. Healthy subjects were thoroughly questioned on their medical history and blood test carefully revised so that any lung, hearth or liver pathology was excluded. Comorbid diseases, such as diabetes mellitus, lung and heart disease, were recorded through a systematic and careful clinical history registration. Similarly, data related to smoking habit were recorded. Glomerular filtration rate was estimated through the “Modification of Diet in Renal Disease” (MDRD) formula. Body Mass Index (BMI) was calculated for patients with chronic hepatitis, but not for cirrhotic patients due to its lack of significance in patient with decompensated liver disease (43 on 65 cirrhotic patients, 66.2%). Hepatic encephalopathy was assessed according to the West-Haven criteria^18^.

Different aetiologies were distinguished by the positivity of HBsAg and anti-HCV antibodies or autoantibodies suggestive of autoimmune liver disease (ANA, ASMA, antiLKM), referred chronic assumption of alcoholic beverage (more than 20 g per day for females and 30 g per day for males) and presence of metabolic comorbidities, such as diabetes mellitus, hypercholesterolemia and hypertriglyceridemia. The Child-Pugh class (CPC) was assessed for all subjects with LC. The study and all methods were approved by our local Ethics Committee (Campus Bio-Medico University of Rome) in accordance with the approved guidelines and all the study participants provided written informed consent.

### Breath collection and delivery

Breath collection was obtained at morning with patients fasting and smoking free for at least 12 hours. Each patient was asked to breath at tidal volume for three minutes into a dedicated storage device for direct sampling of exhaled breath onto adsorbing cartridge (Pneumopipe^®^, European patent n. 12425057.2, Rome - Italy). The adsorbent cartridge used in this work is the Tenax GR, by Supelco^19^. The exhaled breath collected onto cartridges is desorbed into the sensors chamber by an interfacing device able to uniformly heat the tube from 50 °C to 200 °C, and finally cleaning the cartridge holding the temperature at 300 °C for five minutes.

### Sensors

The gas sensor array (also called e-nose) used in this study is the Bionote^20^. It is based on an array of seven quartz microbalances (oscillating at a resonance frequency of 20 MHz) functionalized by a combination of anthocyanins extracted by three different plant tissues: red rose, red cabbage, blue hortensia. The sensors composing the array are: sensor 1, red rose extract 65 mM; sensor 2, blue hortensia extract 65 mM; sensor 3, red cabbage extract 65 mM; sensor 4, red rose extract 65 mM + sucrose 10 mM; sensor 5, red cabbage extract 65 mM + sucrose 10 mM; sensor 6, blue hortensiaextract 65 mM + sucrose 10 mM; sensor 7, sucrose 10 mM. The system is controlled by an electronic board based on a STM 32 F303VC (ST Microelectronics; Geneva, Switzerland). A calibration study on Bionote is reported elsewhere^20^. VOCs extracted from the cartridges at four different temperatures bind to the seven different anthocyanins, inducing a frequency shift of the respective quartz from the reference value and this is registered as the sensor response. The final fingerprint of the exhaled breath (BP) is composed of 28 responses, given by the 7 sensors’ outputs at four different temperature (50–100–150–200 °C).

BPs have been represented with radar-plots; each radar-plot is formed by equi-angular radii on a circumference, where each radius represents one of the 28 sensor responses. Magnitude of each sensor response is given by the radius length. The BP profile consists of a line drawn connecting the data values for each radius on the radar plot.

From each radar plot, the area under the BP profile (AUBP) has been derived as the area enclosed by the BP profile,i.e. as the sum of the areas (*A*_*i*_) of the triangles defined by the BP profile with the radii representing the responses of two consecutive sensors (*s*_*i*_ and *s*_*i*+1_) plus the area (*A*_*0*_) of ​​the triangle bounded by the radii of the first (*s*_1_) and the last sensor (*s*_*n*_). The following formula has been used:


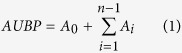



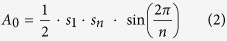



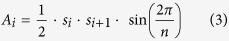


*n* is the number of sensors (28)

*s*_*i*_ is the response value of the *i*^*th*^ sensor

Further technical specifications can be found in the Supplementary File.

### Statistical Method

A Partial Least Square Discriminant Analysis (PLS-DA) has been performed on the 7-dimensional data array and it was used as a regression method for calculating to which extent BP could predict different study groups mentioned above. The PLS-DA models have been cross-validated by 10-fold resampling and 10-fold random permutation of class label.

ROC analysis was used to evaluate the diagnostic performance of continuous variables, that was expressed as AUROC. The coordinates of ROC curves were used to identify the best cut-off values able to separate the compared groups. The replication stability of ROC curves was analysed using 10-fold resampling procedure and 10-fold random permutation of class labels. Mean AUROCs after 10 resamplings and after 10 permutations were computed to test the robustness of our results. Confidence intervals for AUROC were calculated with bootstrap procedure.

The accuracy of diagnostic tests was established with calculation of sensitivity, specificity, positive likelihood ratio (LR+), negative likelihood ratio (LR−), positive predictive value (PPV) and negative predictive value (NPV).

Data are described as means ± standard deviations or as numbers and percentages. Comparisons among groups were assessed by ANOVA test or χ^2^ test as appropriate. The Spearman rho has also been calculated as a measure of correlation between VOC patterns derived by selected sensors and individual liver function tests. PLS-DA have been performed using the PLS-Toolbox SW (Eigenvector, Wenatchee, WA, USA) in the Matlab environment (The Mathworks, Natick, MA, USA). ROC analysis with resampling and permutation procedures were carried out with R Statistical Software version 2.14. Other statistical analysis were performed by using SPSS software (version 21.00; SPSS Inc., Chicago, IL, USA). Statistical significance was assumed at p < 0.05.

## Results

### Patient characteristics

Fifty-six healthy subjects, 39 patients with NC-CLD and 65 patients with LC (CPC A/B/C 21/27/17 patients) were enrolled in the study. Demographics and clinical features of the study groups are presented in Table 1. Mean age was 61.1 ± 12.9 years and 58.1% (93 subjects) were males. As expected, participants with LC were older (63.6 ± 11.5 *vs* 56.1 ± 13.6 years, p < 0.01) than those with NC-CLD and they had higher value of serum bilirubin (2.6 ± 2.8 *vs* 0.6 ± 0.3 *vs* 0.8 ± 0.5 mg/dL, p < 0.01) and higher prevalence of diabetes mellitus (41.5% *vs* 7.1% and 12.8%, p < 0.001) than healthy controls and NC-CLD, respectively. Furthermore, patients with LC and NC-CLD showed a significantly different prevalence of the different aetiologies of CLD (see Table 1). Overall, 8 patients were affected by COPD (1 in NC-CLD group and 7 in LC group, p = ns). No patients with congestive heart failure were enrolled.

### BP pattern models and Areas under BP profile (AUBP)

PLS-DA was performed to build 3 discrete models: 2 three-class models to discriminate LC from NC-CLD and healthy controls and to distinguish different CPC (CPC A, B and C) among LC patients and 1 two-class for the distinction of infective from non-infective (alcoholic, metabolic and autoimmune) hepatopathy.

**Figure 1:**
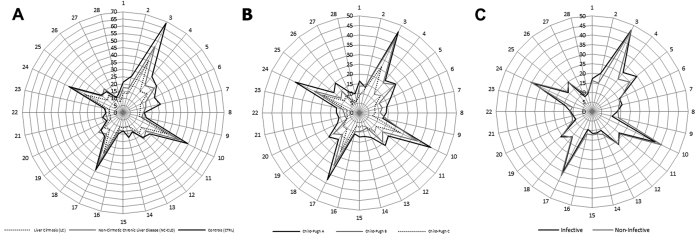
Comparison of BPs relative to patients with LC and NC-CLD and CTRL (Panel A), cirrhotic patients with different CPC (Panel B) and patients with infective and non-infective CLD (Panel C). The radar-plots (see description in the text) reveal a progressive concordant reduction of mean responses from CTRL and NC-CLD to LC patients and, among LC patients, for increasing CPC. Concerning discrimination between infective and non-infective aetiology, major reductions of mean responses have been observed in sensors 2, 4 and 5 at 50 °C for patients with non-infective liver disease.

Radar-plots of patients belonging to different groups are shown in Fig. 1. Visual analysis of radar-plot profiles showed a progressive concordant reduction of mean responses for each sensor from healthy controls and NC-CLD to LC patients (Fig. 1, Panel A) and, among LC patients, for increasing CPC (Fig. 1, Panel B); no discordant directional changes were observed. Mean BPs of healthy controls and of patients with NC-CLD were diffusely wider than those observed for patients with LC, while substantially overlapping in different parts of the BP profile between them; major reduction of sensors’ responses between them can be observed mainly from sensors 1 to 7 at 50 °C (Fig. 1, Panel A).

Area under breath-print profile (AUBP) for each group resulted to be significantly reduced with the development of LC and the progression of hepatocellular failure (1810.3 ± 629.6 for healthy controls *vs* 1349.7 ± 500.6 for NC-CLD *vs* 887.3 ± 478.7 for LC, p < 0.01; 1090.8 ± 288.5 for CPC A *vs* 864.6 ± 509.7 for CPC B *vs* 658.7 ± 478.7 for CPC C, p = 0.02).

Concerning the discrimination between infective and non-infective aetiology, visual analysis of BP profiles revealed less evident differences with major reductions of mean responses in sensors 2, 4 and 5 at 50 °C for patients with non-infective liver disease (Fig. 1, Panel C). AUBP were not significantly different (1070.9 ± 676.2 for infective *vs* 1081.6 ± 450.2 for non-infective, p = 0.92).

To note, while predictive models for LC detection and CPC discriminations were established on the whole population, discriminative model for infective *vs* non-infective disease could be obtained only excluding patients with hepatic encephalopathy.

The accuracy of PLS-DA models were then tested for their classificatory properties (confusion matrix are shown in Tables 2, 3 and 4), showing a sensitivity, specificity, LR+, LR-, PPV and NPV

–for CLD (LC *plus* NC-CLD) vs healthy controls of 86.2%, 98.2%, 48.3, 0.1, 98.3%, 85.9%;

–for LC *vs* NC-CLD, among patient with CLD, of 87.5%, 69.2%, 2.8, 0.2, 80.3% and 79.4%;

–for CPC A-B *vs* C, among patients with LC, of 87.5%, 64.7%, 2.5, 0.2, 87.5% and 64.7%;

–for infective *vs* non-infective liver disease of 29.0%, 88.0%, 2.4, 0.8, 60.0% and 66.7, respectively.

By applying these models to our study population, 16 (24.6%) cirrhotic patients and 12 (30.8%) patients with NC-CLD got misclassified. Groups of well- and mis-classified patients were then compared for major demographic, bioumoral and clinical features, but none of them was found to distinguish the aforementioned groups (Table 5).

### Receiver Operating Characteristic (ROC) analysis of AUBP

**Figure 2:**
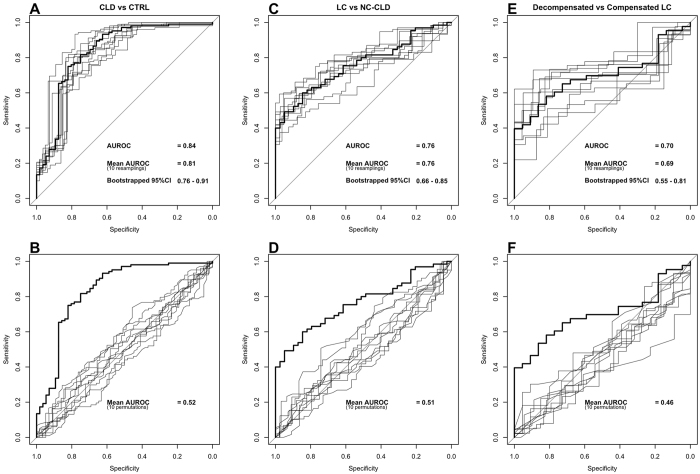
ROC curves (thick lines) with 10 ROC curves (thin lines) for 10 resampling procedures and 10 random permutations of class labels for the predictive performances of the area under BP profile (AUBP) for detenction of: panels **A**,**B** chronic liver disease (CLD) vs healthy controls (CTRL); panels **C**,**D** liver cirrhosis (LC) vs non-cirrhotic chronic liver disease (NC-CLD) in patients with chronic liver disease; panels **E**,**F** decompensated liver cirrhosis (CPC **B**,**C** vs CPC A) in LC patients.

ROC analysis was performed to assess the diagnostic performance of AUBP with regard to CLD, LC and decompensated LC (Fig. 2). These models showed an AUROC of 0.84 (bootstrapped 95% CI 0.76–0.91) for detection of CLD *vs* healthy controls, of 0.76 (bootstrapped 95% CI 0.66–0.85) for distinction of LC *vs* NC-CLD, among patients with CLD, and of 0.70 (bootstrapped 95% CI 0.55–0.81) for detection of decompensated LC. Similar results were obtained also after 10-fold resamplings (mean AUROC 0.81, 0.76 and 0.69, respectively - Fig. 2 panel A-C-E), while significantly lower AUROCs were obtained after 10-fold random permutations of class labels (mean AUROC 0.52 with p < 0.001, 0.51 with p < 0.001 and 0.46 with p < 0.05, respectively - Fig. 2 panel B-D-F), indicating the robustness of the predictive properties of our index. Using the coordinates of ROC curves, the following cut-off values of AUBP were identified: <1248 with sensitivity 65.4%, specificity 87.5%, LR+ 5.2, LR − 0.4, PPV 90.7% and NPV 57.6% for detection of CLD *vs* healthy controls; <862 with sensitivity 43.1%, specificity 97.4%, LR + 16.8, LR − 0.6, PPV 96.6% and NPV 50.7% for diagnosis of LC *vs* NC-CLD; <812 with sensitivity 53.5%, specificity 86.4%, LR + 3.9, LR − 0.5, PPV 88.5% and NPV 48.7% for decompensated liver disease (CPC B-C vs A) in LC patients.

### Sensor correlation with liver function test

As reported in Table 6 significant correlations between response values of the 7 sensors at 4 temperatures were found with serum markers of liver failure (serum bilirubin, albumin and INR). Serum bilirubin showed significant negative correlations with all sensors, except for sensor 2 at 50 °C. Accordingly, negative correlations were found for INR, except for sensor 2 and 6 at 50 °C, sensor 6 at 150 °C and sensor 6 and 7 at 200 °C. On the contrary, serum albumin showed positive correlations with few sensors (2, 3, 4, 5) at 150 °C and only with sensor 5 at 200 °C.

## Discussion

In this study BP analysis by e-nose was used for the first time to analyse exhaled breath of patients with CLD aiming at verifying its discriminative and classificatory properties.

Our findings show that distinctive BPs characterize healthy controls, NC-CLD and LC and, within LC, different stages of hepatocellular failure, as represented by CPC worsening. As secondary outcome, we found BP differences between infective and non-infective (alcoholic, metabolic and autoimmune) liver diseases.

Other scientific works^7^,^8^,^9^,^10^,^11^,^12^,^13^ explored the exhaled breath pattern of patients with CLD using mainly GC/MS or IMS, instead of gas sensor array, i.e. e-nose. Among those, Van der Velde *et al.* found 4 VOCs discriminating LC patients from healthy controls^7^ and, later, Morisco *et al.* documented 12 VOCs associated, not only with LC, but also with CPC^8^. Our results are substantially in line with these data, but we add to these observations by showing that exhaled breath analysis by e-nose identifies well distinguished BPs, that can discriminate patients with LC, not only from healthy controls (as also demonstrated by Morisco *et al.* and Van der Velde *et al.* with analytical techniques^7^,^8^), but also from patients with NC-CLD. Interestingly, patients with NC-CLD retain distinctive BPs from healthy controls, likely reflecting systemic influences of hepatic inflammation or fibrosis underlying NC-CLD.

To note, our findings were very recently confirmed by another study^21^ which analysed the exhaled breath of 87 patients with NC-CLD and 34 LC patients with GC-MS reaching similar classificatory performances with the isolation of a set of 11 discriminatory VOCs.

AUBP has been derived from the graphical representation of BPs on radar charts and has been proposed for the first time as a BP-derived measurable index to further classify patients with CLD (Fig. 2): selected cut-off values resulted to be more accurate for detection of LC, among patients with CLD, and decompensated LC compared to BP analysis by PLS-DA; conversely, BP showed higher classificatory performances in models including healthy controls. Indeed, by applying the PLS-DA model including healthy controls and patients with CLD, CLD could be correctly predicted in the 98.3% (PPV) of cases; accordingly, AUBP cut-off values of 862 and 812 could predict LC and decompensated LC with high accuracy (PPV 96.6% and 88.5%, respectively).

Since liver plays a key role in metabolism, it is easily comprehensible how even early stages of hepatocellular failure or the formation of porto-systemic veno-venous shunts, secondary to portal hypertension, can alter the blood concentration of various endogenous compounds.

These metabolic changes were paralleled by concordant reductions of mean response for each sensor by the e-nose, which were found to be strictly related to liver function tests; in particular, inverse correlations have been evidenced with serum bilirubin and INR (Table 6). Conversely, as pointed out also by Morisco *et al.*^8^, serum albumin seems to be relatively unrelated to VOCs alterations.

Altogether, the demonstration that the complex metabolic modifications underlying CLD and hepatic failure correspond to distinctive BPs might have important practical implication for diagnostic and prognostic purposes: BP measurement might qualify as simple and inexpensive estimate of liver function, favouring the diagnosis of LC and the periodic follow-up of patients with CLD. Furthermore, the AUBP could be a valid index for discriminating LC and classifying patients through different CPC.

As this was the first exploratory study on the use of e-nose in patients with CLD, our data possibly suggest that e-nose could be able to give indications concerning the aetiology of liver disease, in particular if infective or non-infective. Unfortunately, our model showed rather weak performance rates and visual analysis of radar plot evidenced isolated differences in the BP profiles. Furthermore, this discrimination was obtained only after exclusion of patients with hepatic encephalopathy, because they did not conform to a predictable model. It could be speculated that this was likely due to wide metabolic derangements underlying hepatic encephalopathy and to the great phenotypic variability among different types of hepatic encephalopathy (minimal, episodic, recurrent and persistent)^18^, possibly accounting for heterogeneous BP patterns misleading aetiological discrimination. Anyway, the present study was not designed specifically to explore the aetiological aspect, for this reason forthcoming research should therefore better clarify this point.

In the present study BIONOTE e-nose was used to detect VOCs in the exhaled air and the related data matrix was analysed through PLS-DA in order to find the most discriminatory patterns between classes. In the field of breathomics, many other methodological alternatives are available with regard to both measurement techniques (such as the aforementioned GC/MS, IMS/MCC or PTR-MS) and data analysis techniques, such as decision trees, neural networks, random forest, support vector machines. All these methods have got their own strengths and limitations, described elsewhere^22^,^23^,^24^, but they all aim at identifying groups of VOCs and associating them with specific pathologic scenarios.

Concerning e-nose, this technology does not allow to characterize individual VOCs and, then, to interpret the BPs. Anyway, according to a previous calibration study 52 compounds grouped into 5 families have been identified to be typically desorbed from the Tenax tube at 4 different temperatures^25^. However, as previously explained (see Fig. 1), we have found a widespread concordant reduction of sensors’ responses at all temperatures between healthy controls, NC-CLD and LC patients and, among LC patients, between CPC. Thus, only a quantitative variation in VOCs seems to characterize this evolution. Conversely, the differences observed for sensors’ responses at 50 °C between infective and non-infective liver diseases suggests different concentrations of the first class of compounds to be relevant for the aetiological diagnosis.

In a previous study by Incalzi *et al.* on patients with obstructive sleep apnoea syndrome (OSAS), basal BP and BP change after the first night of continuous positive airways pressure were found to be strictly related to the burden of comorbid diseases, particularly those impacting the metabolism such as metabolic syndrome, diabetes mellitus or congestive heart failure^26^. At variance with this latter study, the comorbidity analysis of the 16 cirrhotic patients and of the 12 patients with NC-CLD, that resulted misclassified by our PLS-DA model, has not shown differences in the prevalence of major comorbidities compared to correctly classified ones (Table 5). To note, the two study populations were fairly different both for basal pathologies, namely CLD and OSAS, and for prevalence of comorbidities, as we did not include patients with congestive heart failure and the OSAS population was free from renal failure^26^. Anyway, well-designed studies aiming at assessing whether and to which extent BP patterns of selected diseases depend upon comorbidity are still eagerly awaited.

This study has some limitations which need to be solved in our future analysis of patients with CLD. First of all, the study population should be expanded and well-represented groups according to diagnosis separately analysed. Second, the classificatory role of comorbidity would be better defined by integrating the list of comorbid diseases with some index of severity. Third, although exhaled breath analysis could be performed for both diagnostic and prognostic purposes, this study cared only the diagnostic and classificatory aspects; studies on the prognostic properties of this analysis should necessarily follow. The lack of a testing population is a further limit of our study, which accordingly qualifies as a proof of concept study. However, to test the reproducibility of our results PLS-DA models and the ROC curves were cross-validated through 10-fold resampling and permutations, which testified to the robustness of our classificatory model.

This study has also its strength. First, this is one of the biggest study analysing exhaled breath pattern in patients with CLD and it is the first time that the e-nose is used in this setting. Second, it deals with a real life population and it relies upon an innovative standardized non-invasive technology for breath sampling, storage and delivery. Third, while previous studies simply compared LC patients with healthy controls, our results demonstrate that the e-nose is able to discriminate LC from NC-CLD and NC-CLD from healthy controls. Then, sensitivity, specificity, LRs and predictive values should be interpreted and weighted accordingly.

In conclusion, these data are proof of concept that the e-nose could be a valid non-invasive instrument to characterize liver disease, in terms of evolution into LC or aetiology, and monitor hepatic function through a qualitative analysis of BP or, conceivably, a quantitative analysis of derived indexes, such as the AUBP. To note, VOC analysis by e-nose is easy to perform, time sparing and also economically competitive. The observed classificatory properties might be further improved by refining stage-specific BP and considering the impact of comorbidities in a larger series of patients.

## Additional Information

**How to cite this article**: De Vincentis, A. *et al.* Breath-print analysis by e-nose for classifying and monitoring chronic liver disease: a proof-of-concept study. *Sci. Rep.* **6**, 25337; doi: 10.1038/srep25337 (2016).

## Supplementary Material

Supplementary Information

## Figures and Tables

**Table 1:** Demographics and clinical features of the study population.

	CTRL	NC-CLD	LC	p
N (%)	56(35)	39(24.4)	65(40.6)	–
Age (years), mean ± SD	64.9 ± 11.0	56.1 ± 13.6*	63.6 ± 11.5 §§	<0.01
Male, n (%)	29 (51.8)	18 (46.2)	46(70.8)*§	<0.05
Child-Pugh ClassA, n(%)/B, n(%)/C, n(%)	–	–	21(32.3)/27(41.5)/17(26.2)	–
Aetiology				
Infective, n (%)	–	19(48.6)	18(27.7)	<0.01
Alcoholic, n (%)	–	1(2.6)	17(26.1)	<0.01
Metabolic, n (%)	–	17(43.6)	13(20.0)	<0.01
Autoimmune, n (%)	–	1(2.6)	3(4.7)	0.02
Infect/Alcoh, n (%)	–	0(0.0)	9(13.8)	<0.01
Infect/Metab, n (%)	–	0(0.0)	1(1.6)	<0.01
Alcoh/Metab, n (%)	–	1(2.6)	4(6.1)	<0.01
Diabetes Mellitus, n (%)	4(7.1)	5(12.8)	27(41.5)**§§	<0.01
ALT (U/L), mean ± SD	25.1 ± 7.9	52.3 ± 48.7**	52.2 ± 42.9**	<0.01
Bilirubin (mg/dL), mean ± SD	0.6 ± 0.3	0.8 ± 0.5	2.6 ± 2.8**§§	<0.01
eGFR (mL/min), mean ± SD	87.5 ± 26.0	84.6 ± 18.7	83.6 ± 36.4	ns
Current Smoker, n (%)	9(14.3)	9(23.1)	18(27.7)	ns
BMI (kg/m^2^), mean ± SD	26.0 ± 3.4	27.9 ± 4.3	−^a^	ns
Lung disease, n (%)	0(0.0)	1(2.6)	7(11.2)*	<0.05
AUBP, mean ± SD	1810.3 ± 629.6	1349.7 ± 500.7**	887.3 ± 478.7**§§	<0.01

CTRL, healthy controls; NC-CLD, non-cirrhotic chronic liver disease; LC, liver cirrhosis; difference between groups were calculated with ANOVA test for continuous variables or with χ^2^ test for categorical variables; Bonferroni correction was used for multiple testing; *and **, p < 0.05 and p < 0.01 compared to CTRL; ^§^and ^§§^, p < 0.01 and 0.05 compared to NC-CLD; ^a^not calculated due to its lack of significance in patient with decompensated liver disease (43 on 65 cirrhotic patients, 66.2%); ALT, Alanine Transaminase; eGFR, estimated glomerular filtration rate; BMI, body mass index.

**Table 2:** Confusion matrix for classification of liver cirrhosis (LC), non-cirrhotic chronic liver disease (NC-CLD) and healthy controls (CTRL).

		Predicted		
		LC	NC-CLD	CTRL
Actual	LC	49	7	9
	NC-CLD	12	27	0
	CTRL	0	1	55

**Table 3:** Confusion matrix for classification of Child-Pugh class (CPC) A, B and C.

		Predicted		
		CPC A	CPC B	CPC C
Actual	CPC A	15	4	3
	CPC B	8	15	3
	CPC C	4	2	11

**Table 4:** Confusion matrix for classification of Infective and Non-Infective liver disease.

	Predicted		
		Infective	Non-Infective
Actual	Infective	9	22
	Non-Infective	6	44

Data obtained after exclusion of 10 patients with mixed aetiology (infective-alcoholic and infective-metabolic) and, then, of 13 patients with hepatic encephalopathy.

**Table 5:** Demographics and clinical features of patients with non-cirrhotic chronic liver disease(NC-CLD) and liver cirrhosis (LC) and within groups of well-classified or misclassified patients by Partial Least Square Discriminant Analysis (PLS-DA) models.

	All		Well-classified		Misclassified		p*
	NC-CLD	LC	NC-CLD	LC	NC-CLD	LC	
N (%)	39	65	27(69.2)	49(75.4)	12(30.8)	16(24.6)	–
Age (years), mean ± SD	56.1 ± 13.6	63.6 ± 11.5	57.1 ± 14.2	65.0 ± 9.6	51.8 ± 21.3	59.1 ± 15.1	ns/ns
Male, n (%)	18(46.2)	46(70.8)	12(44.4)	34(69.4)	6(50.0)	12(75.0)	ns/ns
Child-Pugh class A/B/C	–	21/27/17	–	17/22/10	–	4/5/7	ns/ns
Diabetes mellitus, n (%)	5(12.8)	27(41.5)	3(11.1)	20(40.8)	2(16.7)	7(43.7)	ns/ns
ALT (U/L), mean ± SD	52.3 ± 48.7	52.2 ± 42.9	53.6 ± 41.8	48.7 ± 34.7	37.8 ± 24.1	54.5 ± 58.8	ns/ns
Bilirubin (mg/dL), mean ± SD	0.8 ± 0.5	2.6 ± 2.8	0.6 ± 0.2	2.3 ± 2.0	1.2 ± 0.8	2.8 ± 2.1	ns/ns
eGFR (mL/min), mean ± SD	84.6 ± 18.7	83.6 ± 36.4	82.7 ± 20.6	83.3 ± 35.6	84.2 ± 14.9	84.5 ± 39.6	ns/ns
Smokers, n (%)	9(23.1)	18(27.7)	6(22.2)	13(26.5)	3(25.0)	5(31.3)	ns/ns
Lung disease, n (%)	1(2.5)	7(10.8)	1(3.7)	4(8.2)	0(0)	3(18.7)	ns/ns

Difference between groups were calculated with ANOVA test for continuous variables or with χ^2^ test for categorical variables; Bonferroni correction was used for multiple testing; *p value for differences between well- and misclassified groups of patients with NC-CLD and LC, respectively; NC-CLD, non-cirrhotic chronic liver disease; LC, liver cirrhosis; ALT, Alanine Transaminase; eGFR, estimated glomerular filtration rate; ns, not significant (p > 0.05).

**Table 6:** Spearman’s ρ correlation coefficient between sensors and liver function tests.

	T = 50 °C						T = 100 °C						T = 150 °C						T = 200 °C					
	Bilirubin		Albumin		INR		Bilirubin		Albumin		INR		Bilirubin		Albumin		INR		Bilirubin		Albumin		INR	
	ρ	p	ρ	p	ρ	P	ρ	p	ρ	p	ρ	p	ρ	p	ρ	p	ρ	p	ρ	p	ρ	p	ρ	p
Sens 1	−0.5	<0.01	–	ns	−0.4	<0.01	−0.5	<0.01	–	ns	−0.4	<0.01	−0.4	<0.01	–	ns	−0.3	<0.01	−0.4	<0.01	–	ns	−0.3	0.02
Sens 2	–	ns	–	ns	–	Ns	−0.3	0.01	–	ns	−0.3	0.04	−0.3	<0.01	0.4	<0.01	−0.3	0.01	−0.4	<0.01	–	ns	−0.3	0.03
Sens 3	−0.4	<0.01	–	ns	−0.3	<0.01	−0.5	<0.01	–	ns	−0.4	<0.01	−0.5	<0.01	0.3	0.05	−0.4	<0.01	−0.5	<0.01	–	ns	−0.3	0.01
Sens 4	−0.4	<0.01	–	ns	−0.3	<0.01	−0.5	<0.01	–	ns	−0.4	<0.01	−0.5	<0.01	0.3	0.05	−0.3	0.01	−0.4	<0.01	–	ns	−0.3	0.03
Sens 5	−0.4	<0.01	–	ns	−0.3	<0.01	−0.5	<0.01	–	ns	−0.4	<0.01	−0.5	<0.01	0.3	0.04	−0.4	<0.01	−0.4	<0.01	0.3	0.04	−0.3	0.02
Sens 6	−0.3	<0.01	–	ns	–	Ns	−0.4	0.01	–	ns	−0.3	0.02	−0.2	0.02	–	ns	–	ns	−0.3	0.01	–	ns	–	ns
Sens 7	−0.5	<0.01	–	ns	−0.4	<0.01	−0.5	<0.01	–	ns	−0.3	0.01	−0.5	<0.01	–	ns	−0.3	<0.01	−0.4	<0.01	–	ns	–	ns

INR, international normalized ratio.
