# Supplementary material for: Breath-print analysis by e-nose for classifying and monitoring chronic liver disease: a proof-of-concept study
Source: Sci Rep. 2016 May 5;6:25337. doi: 10.1038/srep25337 (PMC4857073; doi:10.1038/srep25337)
Supplement: Supplementary Information [file srep25337-s1.doc]

**Title page**

**Breath-print analysis by e-nose for classifying and monitoring chronic liver disease: a proof-of-concept study.**

Antonio De Vincentis^*1^, Giorgio Pennazza^2^, Marco Santonico^2^, Umberto Vespasiani-Gentilucci^1^, Giovanni Galati^1^, Paolo Gallo^1^, Chiara Vernile^2^, Claudio Pedone^3^, Raffaele Antonelli Incalzi^3, 4^ and Antonio Picardi^1^.

*^1^Clinical Medicine and Hepatology Department, Campus Bio-Medico University, via Alvaro del Portillo 200, 00128 Rome, Italy;^2^ Center for Integrated Research - CIR, Unit of Electronics for Sensor Systems, Campus Bio-Medico University, via Alvaro del Portillo 200, 00128 Rome, Italy;^3^ Chair of Geriatrics, Unit of Respiratory Pathophysiology, Campus Bio-Medico University, via Alvaro del Portillo 200, 00128 Rome, Italy; ^4^ San Raffaele- Cittadella della Carità Foundation, Taranto, Italy*

**Supplementary File: Technical specifications for measure chain for exhaled breath collection and analysis.**

Exhaled breath analysis (1), a non-invasive diagnostic procedure, is based upon

- a sampling device for breath collection
- an apparatus for the sample delivery into the measure chamber
- a gas sensor array (BIONOTE eNose).

*Breath collection*

- Breath collection was obtained by Pneumopipe (Supplementary Figure 1), a dedicated storage device for direct sampling of exhaled breath onto adsorbing cartridge (Tenax tube).
- The procedure of breath collection was performed asking each patient to breath at tidal volume for three minutes into the Pneumopipe (Supplementary Figure 2).


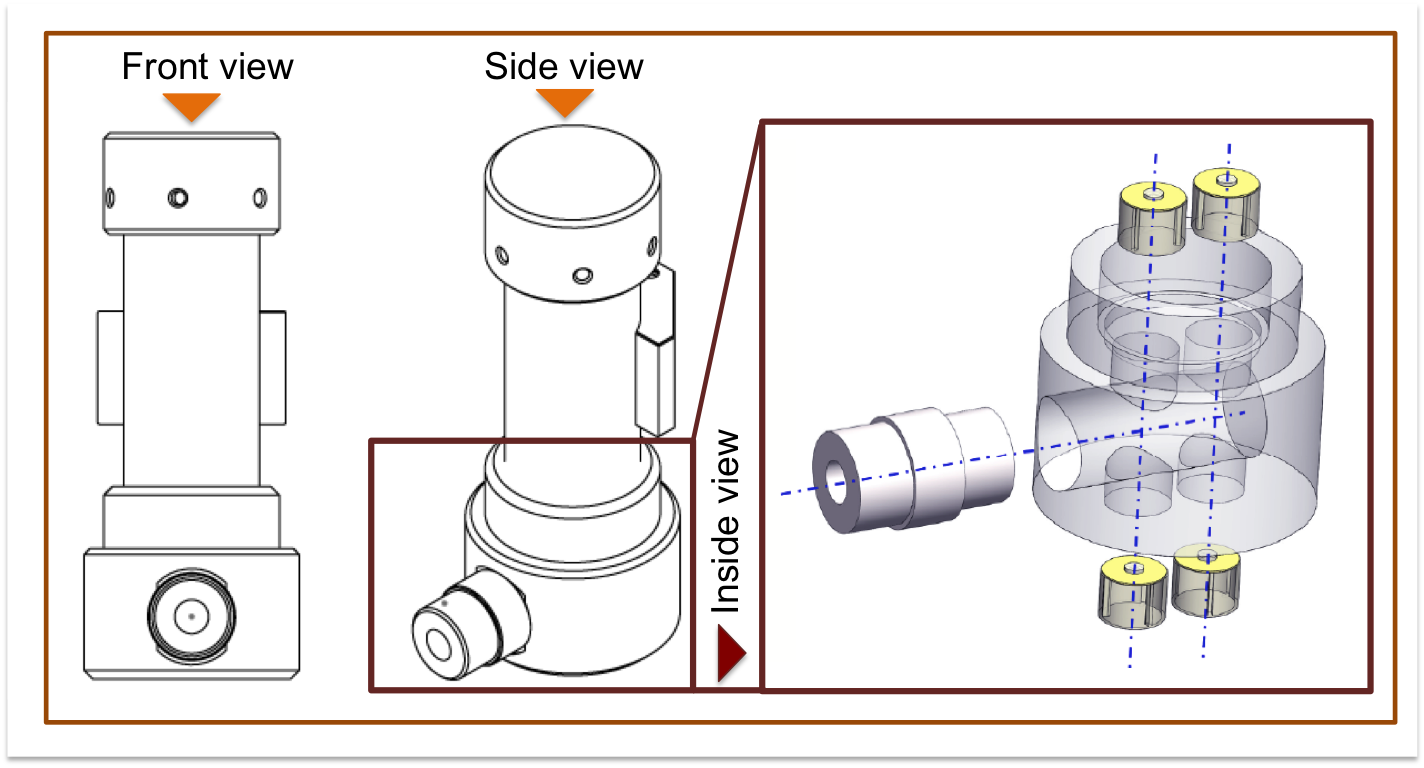

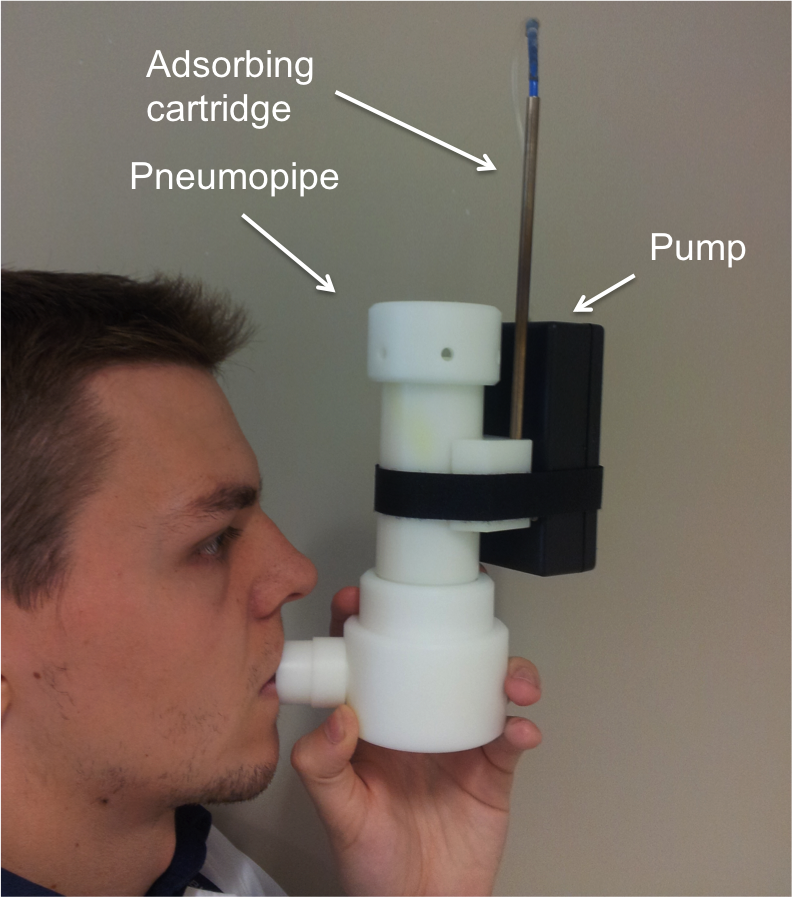


Supplementary Figure 1 Supplementary Figure 2

General overview of Pneumopipe Breath collection via Pneumopipe

- The Pneumopipe, thanks to its double-chamber structure, permitted a continuous sampling of exhaled air, even during an inhalation step.
- Sampling of exhaled breath onto adsorbing cartridge (Tenax tube) was operated by a pump, which sucked the exhaled breath collected in the main chamber of the pneumopipe at a constant flow of 80 ml/min for 3 min (Supplementary Figure 2).

*Breath delivery*

- Breath delivery from the adsorbing cartridge was obtained through an interface-apparatus (Supplementary Figure 3), that was studied with the goal of obtaining a uniform heating of the tube at 50-100-150 and 200°C and finally cleaning the cartridge, holding the temperature at 300°C for five minutes. This means that for each sampling procedure onto the Tenax cartridge, the cartridge content is desorbed in 4 different steps (at each of the aforementioned temperatures) and independently processed into the sensors’ chamber.


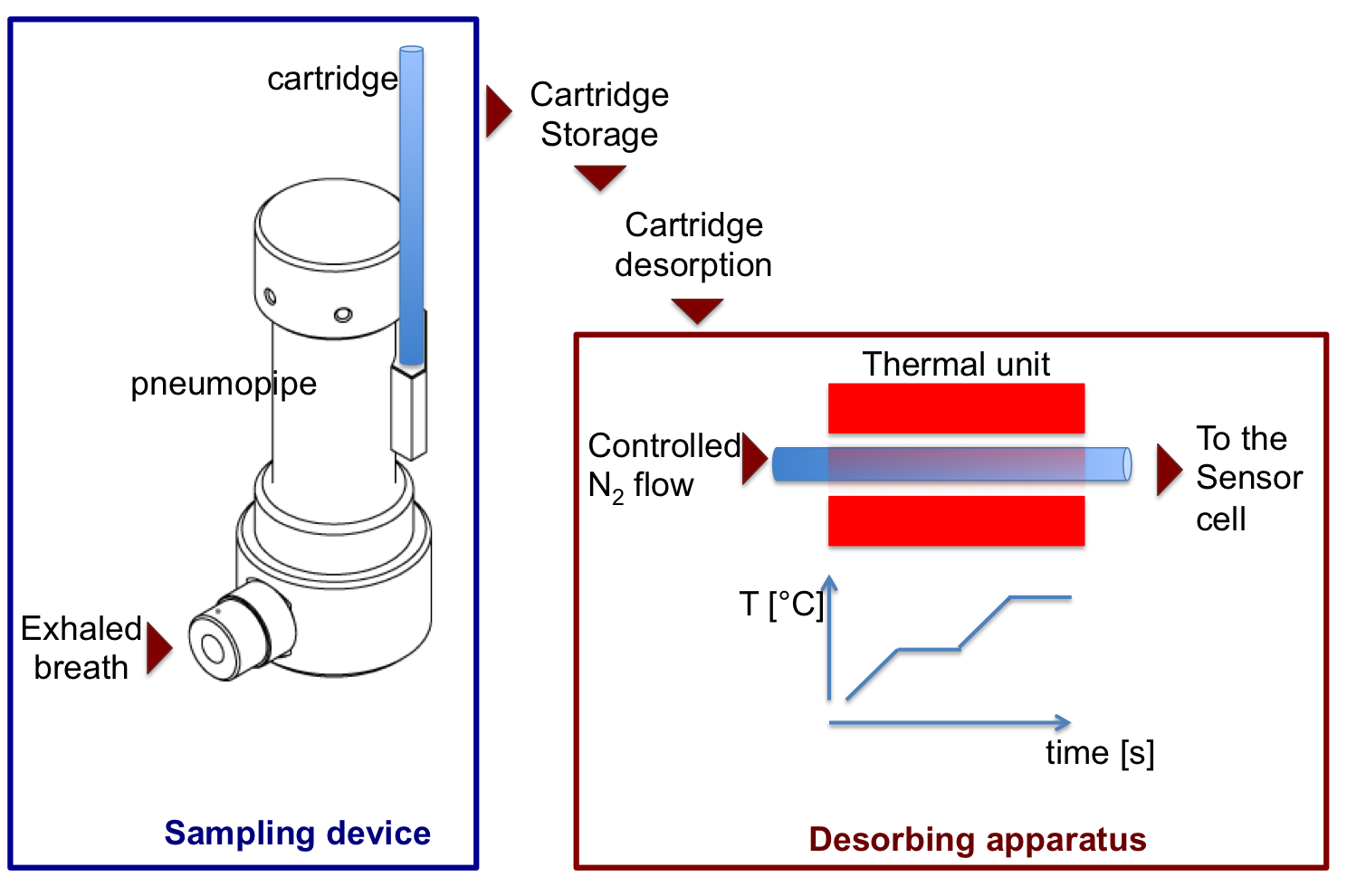


Supplementary Figure 3

General overview of the measure chain for exhaled breath collection and analysis.

*Sensors*

- Sensors consisted of a gas sensor array in which the chemical interactive material was non selective and was intended to imitate the natural olfaction mechanisms, whose olfactive receptors are non selective.
- This array was made up of seven quartz microbalances (piezoelectric crystals) covered by seven different anthocyanins (reported in the text) and owning a fundamental resonance frequency of 20 MHz.
- Once desorbed from the adsorbing cartridge, volatile organic compounds of the exhaled breath chemically bind with different anthocyanins above the seven quartz microbalances of the gas sensor array, inducing a frequency shift from the fundamental resonance frequency. This frequency shift is registered as the sensor response value and is directly proportional to the mass load graving on its surface, as ruled by Sauerbrey [2] and reported below:

$$\Delta f_{0}=-\cdot\frac{2f_{^{0}}^{2}}{A\sqrt{\mu_{q}\rho_{q}}}\Delta m_{s}$$

where A is the coated area, ρ_q_ the quartz density, μ_q_ is the shear stiffness, f_0_ is the fundamental frequency, m_s_ is the quartz mass.

- The final fingerprint of the exhaled breath (breath-print: BP) is a sequence of 28 responses, given by the 7 responses of a 7 dimensional gas senso array at four temperature (50-100-150-200°C). Thus, each sensor gives four responses, one for each temperature.

*Visual representation*

- BPs have been represented with radar-plots (Supplementary Figure 4); each radar-plot is formed by equi-angular radii on a circumference, where each radius represents one of the 28 sensor responses. Magnitude of each sensor response is given by the radius length. The BP profile consists of a line drawn connecting the data values for each radius on the radar plot.


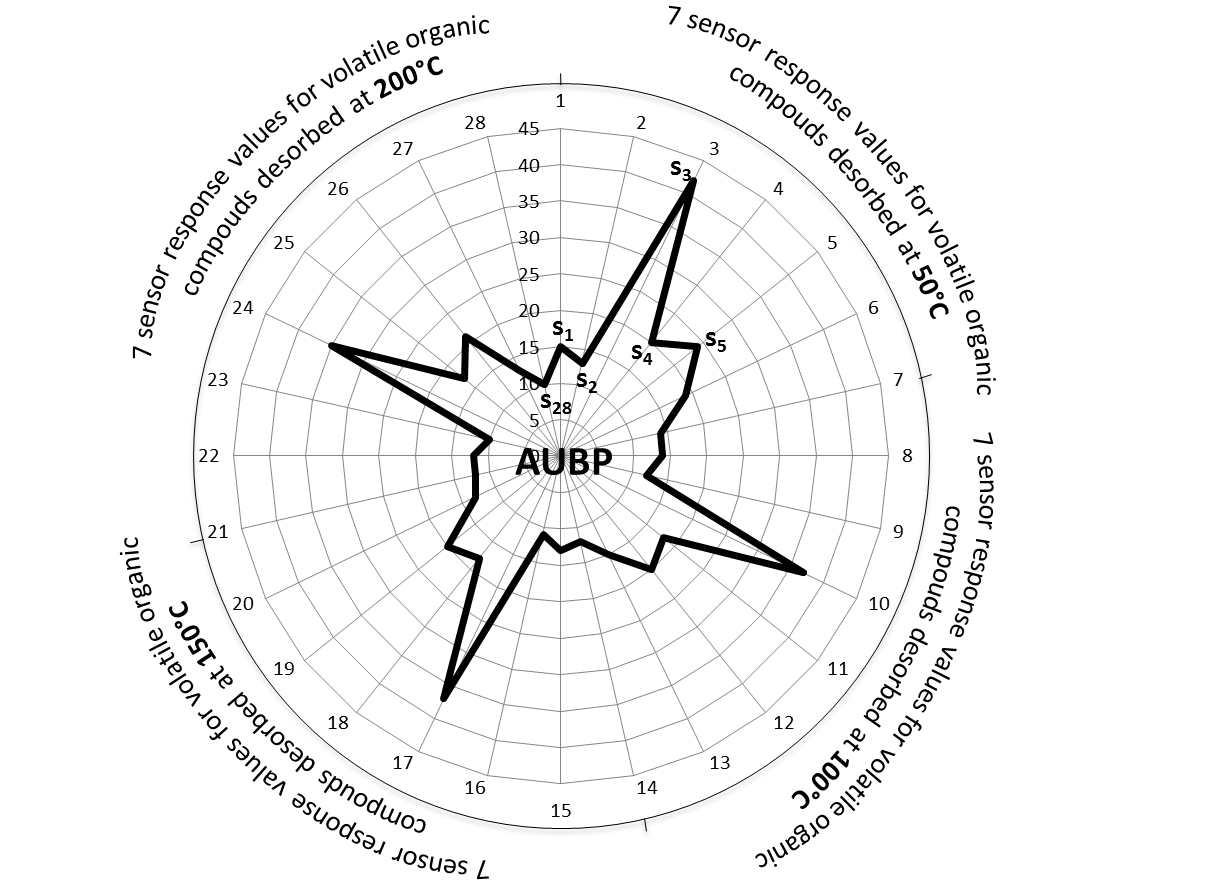


Supplementary Figure 4

Radar plot representing breath-print:

1) 28 sensor responses (7 values for each temperature) are represented on equiangular radii

2) s_1_, s_2_, s_3_…s_28_ represent the magnitude of the response values for each sensor

3) **Black Line** represents the breath print profile obtained connecting the response values on each radius

4) AUBP is represented by the area enclosed by the black line

- The area under the BP profile (AUBP) has been derived as the area enclosed by the BP profile, i.e. as the sum of the areas (A_i_) of the triangles defined by the BP profile with the radii representing the responses of two consecutive sensors (s_i_ and s_i+1_) plus the area (A_0_) of ​​the triangle bounded by the radii of the first (s_1_) and the last sensor (s_28_). The formula, that has been used, is reported in the text.

**References**

[1] Pennazza G et al, Measure chain for exhaled breath collection and analysis: A novel approach suitable for frail respiratory patients. [Sensors and Actuators B Chemical](http://www.researchgate.net/journal/0925-4005_Sensors_and_Actuators_B_Chemical), 2014; 204:578-587

[2] G. Sauerbrey, Use of quartz crystal vibrator for weighing thin films on a microbalance, Zitung Physik, 1959.
